# Comparing mini-sternotomy to full median sternotomy for aortic valve replacement with propensity-matching methods

**DOI:** 10.3389/fsurg.2022.972264

**Published:** 2022-10-10

**Authors:** Rui Liu, Jiangping Song, Junmin Chu, Shengshou Hu, Xian-qiang Wang

**Affiliations:** Cardiac Surgical Department, Chinese Academy of Medical Sciences and Peking Union Medical College Fuwai Hospital, Beijing, China

**Keywords:** mini-sternotomy, aortic valve replacement, MS, FS, cosmetic incision

## Abstract

**Objective:**

This study aims to compare clinical outcomes between mini-sternotomy and full median sternotomy for aortic valve replacement using propensity-matching methods.

**Methods:**

From August 2014 to July 2021, a total of 1,445 patients underwent isolated aortic valve surgery, 1,247 *via* full median sternotomy and 198 *via* mini-sternotomy. To reduce the impact of potential confounding factors, a propensity score based on 18 variables is used to obtain 198 well-matched case pairs, which include 231 aortic valve regurgitations and 165 aortic stenosis cases.

**Result:**

Occurrences of in-hospital mortality (*P* = 0.499), stroke (*P* > 0.999), renal failure (*P* = 0.760), and paravalvular leakage (*P *= 0.224) are similar between the two groups. No significant difference in operation, cardiopulmonary bypass, and aortic cross-clamp times are found between the two groups. However, compared with the full sternotomy group, the mini-sternotomy group has less postoperative 24-hour drainage (131.7 ± 82.8 ml, *P* < 0.001) and total drainage (459.3 ± 306.3 ml, *P* < 0.001). The median mechanical ventilation times are 9.4 [interquartile range (IQR) 5.4–15.6] and 9.8 (IQR 6.1–14.4) in mini-sternotomy and full sternotomy groups (*P* = 0.284), respectively. There are no significant differences in intensive care unit stay and postoperative stay between the two groups. For either aortic valve regurgitations or aortic stenosis patients, significantly less postoperative 24-h and total drainage are still found in the mini-sternotomy group compared with the full sternotomy group.

**Conclusions:**

Mini-sternotomy for aortic valve replacement is a safe procedure, with not only cosmetic advantages but less postoperative drainage compared with full sternotomy. Mini-sternotomy should be considered for most aortic valve operations.

## Introduction

Aortic valve replacement (AVR) is the one of most frequent cardiac surgery in the world. Currently, most AVRs are performed safely with full median sternotomy (FS), but the sternotomy-related complications such as markedly visible midline scar, postoperative pain, and respiratory tract infection make some patients not accept this conventional surgical approach. To reduce surgical trauma, several minimally invasive approaches have been developed for AVR since 1993 and have been associated with optimal outcomes compared with full sternotomy (1–4). The most common minimally invasive approach involves a mini-sternotomy (MS) with less spreading of the incision, not interfering with the diaphragm and less tissue dissection. Many studies have documented that MS may be associated with less bleeding, fewer transfusion requirement, shorter intensive care unit (ICU) and hospital stay despite longer operative, cardiopulmonary bypass (CPB), and cross-clamp times (5–9). Even though definitive clinical evidence is still lacking, the advantages of less pain, less bleeding, and improved cosmesis are generally accepted by patients and cardiac surgeons (10, 11).

Despite these potential advantages of MS for AVR, full sternotomy remains the most widely used approach in isolated aortic valve operation. Therefore, to evaluate the potential benefits of a mini-sternotomy approach, we performed a propensity-matched comparison of short-term outcomes in patients who had mini-sternotomy aortic valve surgery compared with patients who accepted full sternotomy.

## Methods

### Patients selection

**Figure 1:**
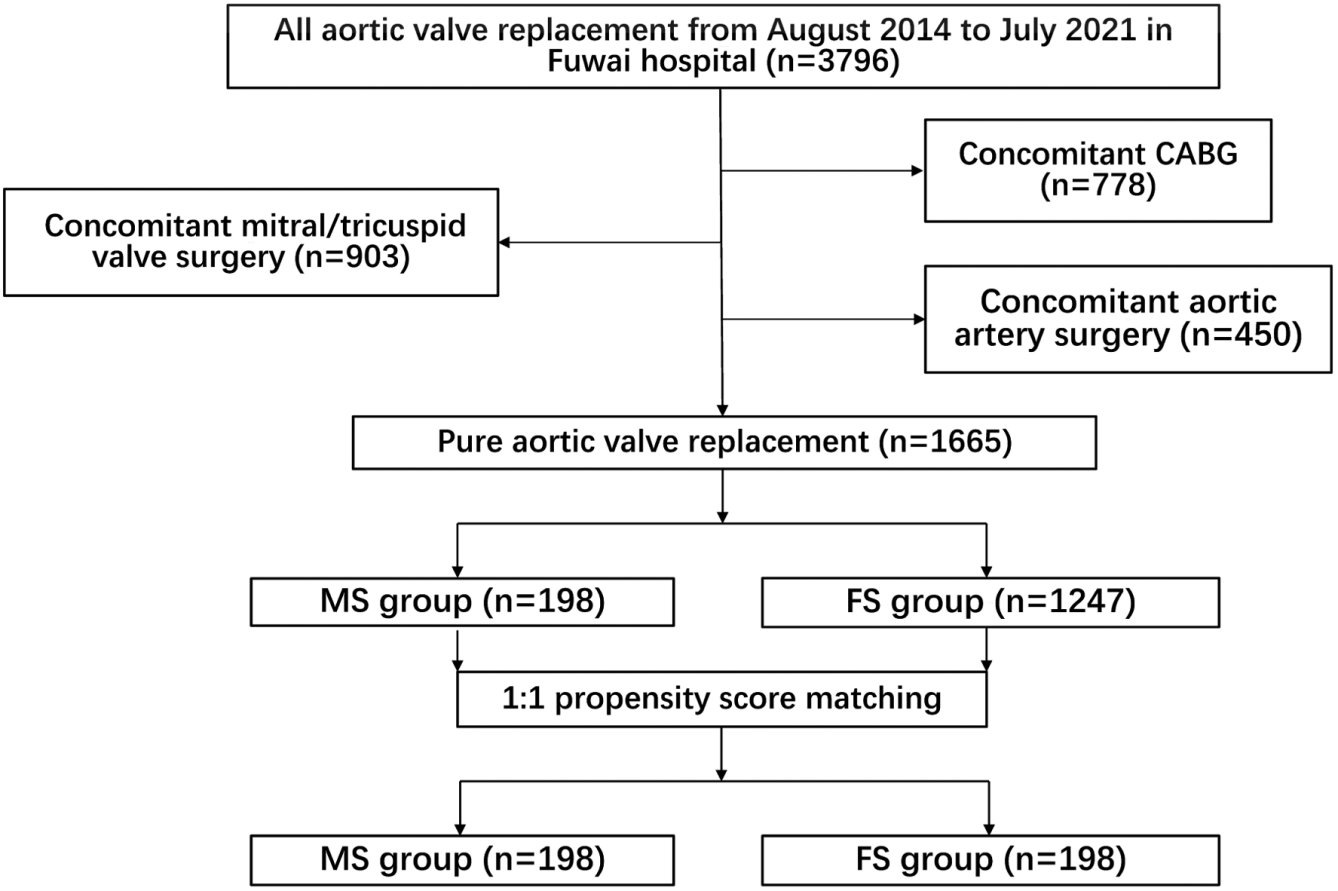
The flow chart of the cohort.

We identified 1,445 patients who underwent isolated aortic valve surgery, including 1,247 patients *via* full median sternotomy and 198 patients *via* mini-sternotomy, from August 2014 to July 2021 at Fuwai Hospital in Beijing. The study protocol was reviewed and approved by the Ethics Committee of Fuwai Hospital on November 24, 2020 (Approval No. 2020-1402). Patients fulfilling these criteria were excluded: (1) Age <18 years; (2) Undergoing concomitant operations, such as mitral valve surgery, ascending aorta replacement, or coronary artery bypass grafting; (3) Active endocarditis. Diagnoses were decided based on echocardiography. Patients are divided into two groups: the FS group (1,247 patients) and the MS group (198 patients). Figure 1 shows the flow chart. Data are retrieved acquired by reviewing each patient's medical records, including operative reports, examination reports, and outpatient clinic notes.

### Surgical technique

**Figure 2:**
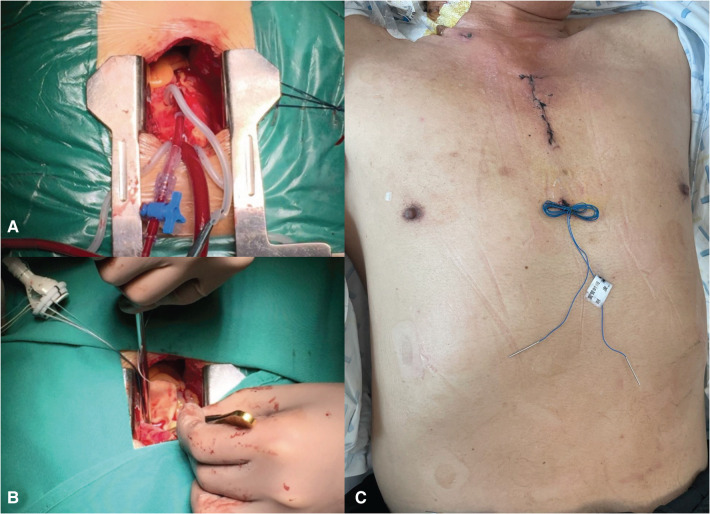
The mini-sternotomy AV replacement procedure. (**A**) The pericardium was opened to expose the ascending aorta, aortic root, and right atrial appendage. The aorta was cannulated conventionally. (**B**) The approach to the aortic valve was *via* an oblique aortotomy carried into the noncoronary cusp above the sinotubular junction. A suitably sized aortic valve prosthesis was inserted using horizontal mattress interrupted sutures. (**C**) Skin was incised from the sternal angle to the level of the fourth intercostal space, approximately 5–6 cm. AV, aortic valve.

All patients included in the study were operated on by surgeons with similar seniority in the same department of cardiovascular surgery. The patients are in the supine position. Skin is incised from the sternal angle to the level of the third intercostal space, approximately 5–7 cm. The manubrium is divided from the suprasternal notch to the right fourth intercostal space forming a J. The pericardium is opened to expose the ascending aorta, aortic root, and right atrial appendage. The aorta is cannulated conventionally and the right atrial appendage is cannulated using a flat venous cannula. After activated clotting time >450 s, CPB is initiated. The ascending aorta is cross-clamped. The approach to the aortic valve is *via* an oblique aortotomy carried into the noncoronary cusp above the sinotubular junction. Cold blood cardioplegia was administered through the coronary ostium. The diseased valve is excised, and a suitably sized aortic valve prosthesis is inserted using horizontal mattress interrupted sutures. After the resumption of heartbeat, ventricular epicardial pacing wires are inserted, and the patient is weaned off CPB. Usually, the pleura is complete, drainage tubes are placed into the anterior mediastinum and posterior pericardial space (Figure 2).

### Statistical analysis

Statistical analysis is performed using SPSS (version 24.0; SPSS Inc., Chicago, IL, United States). Categorical data are presented as numbers (percentage) and continuous variables as means and standard deviations or medians with interquartile ranges (IQRs). A 1:1 propensity score matching (PSM) of FS and MS minimized this potential bias. The propensity scores are estimated with multivariable logistic regression models. Standardized mean differences for all baseline covariates are less than 10% in the matched sample, which suggests a balance of covariates between the FS and MS groups. The matched covariates included gender, age, weight, smoking, aortic valve regurgitation, aortic valve stenosis, history of diabetes, hypertension, previous stroke, atrial fibrillation, chronic renal dysfunction, preoperative lab test (hemoglobin, platelet count, and activated partial thromboplastin time), EUROSCORE, SINOSCORE, preoperative left ventricular end diastolic diameter, and left ventricular ejection fraction. The final PSM sample consists of 198 well-matched pairs of full median sternotomy and mini-sternotomy patients. All patients only accepted isolated aortic valve replacement procedures, including 231 aortic valve regurgitations and 165 aortic stenosis cases. The significant difference is assessed using Pearson's chi-square or Fisher's exact test for categorical variables. Continuous variables are compared using the Student's paired *t*-test or Wilcoxon signed-rank test between the two groups. The *α *<* *0.05 (alpha error level) is considered to be statistically significant.

## Results

### Patient characteristics

The demographic and clinical characteristics of patients are presented in Table 1. Briefly, patients in the FS group were at a significantly younger age than those in the MS group (*P* = 0.033), and patients with mini-sternotomy had more weight than patients with full median sternotomy (*P* = 0.030). Compared with MS group, FS group had a more EUROSCORE [4 (IQR 3–5) vs. 2 (IQR 0–3), *P < *0.001) and SINOSCORE [9 (IQR 7–10) vs. 4 (IQR 4–7), *P < *0.001). There were no statistically significant differences in aortic valve regurgitation, aortic valve stenosis, history of diabetes, hypertension, and previous stroke between the two groups. After PSM adjustment, all clinical covariates were well balanced.

**Table 1:** Baseline characteristics in the overall series and propensity score-matched pairs.

	Overall series			Propensity score-matched			
	FS group (*n* = 1247)	MS group (*n* = 198)	*P* ^a^	FS group (*n* = 198)	MS group (*n* = 198)	*P* ^b^	SMD (%)
Gender (male, %)	897 (71.9)	138 (69.7)	0.517	127 (64.1)	138 (69.7)	0.214	8.7
Age (years)	52.8 ± 14.6	54.9 ± 12.7	0.033	53.2 ± 13.8	54.9 ± 12.7	0.176	9.2
Weight (kg)	68.7 ± 13.6	70.7 ± 12.2	0.030	71.1 ± 14.2	70.7 ± 12.2	0.332	6.4
Smoking	387 (31.0)	78 (39.4)	0.022	72 (36.4)	78 (39.4)	0.604	4.4
AR	717 (57.5)	123 (62.1)	0.251	108 (54.6)	123 (62.1)	0.126	9.8
AS	524 (42.0)	75 (37.9)	0.272	90 (45.5)	75 (37.9)	0.126	9.8
Diabetes	82 (6.6)	10 (5.1)	0.414	11 (5.6)	10 (5.1)	0.654	3.7
Hypertension	463 (37.1)	85 (42.9)	0.118	75 (37.9)	85 (42.9)	0.306	6.8
Previous stroke	1 (0.1)	1 (0.5)	0.135	0 (0.0)	1 (0.5)	0.540	4.7
Atrial fibrillation	29 (2.3)	3 (1.5)	0.609	4 (2.0)	3 (1.5)	0.989	0.7
Chronic renal dysfunction	12 (1.0)	3 (1.5)	0.449	2 (1.0)	3 (1.5)	0.999	0.4
Preoperative lab test							
HB (g/L)	130.6 ± 16.8	132.5 ± 14.8	0.132	131.5 ± 18.2	132.5 ± 14.8	0.454	5.2
PLT count (10^9^/L)	205.1 ± 58.0	202.1 ± 53.0	0.501	203.9 ± 49.4	202.1 ± 53.0	0.653	3.7
APTT(S)	37.8 ± 5.5	36.7 ± 6.2	0.074	37.2 ± 6.5	36.7 ± 6.2	0.214	7.9
EUROSCORE	4 (IQR 3–5)	2 (IQR 0–3)	<0.001	2 (IQR 0–3)	2 (IQR 0–3)	0.864	1.6
SINOSCORE	9 (IQR 7–10)	4 (IQR 2–7)	<0.001	5 (IQR 2–7)	4 (IQR 2–7)	0.223	8.5
LVEDD (mm)	57.4 ± 11.7	57.6 ± 10.3	0.908	56.9 ± 14.2	57.6 ± 10.3	0.863	1.3
LVEF%	59.2 ± 10.1	59.6 ± 8.6	0.713	59.1 ± 9.5	59.6 ± 8.6	0.754	2.5

AR, aortic valve regurgitation; AS, aortic stenosis; LVEDD, left ventricular end diastolic diameter; LVEF, left ventricular ejection fraction; SMD, standardized mean difference; HB, hemoglobin; PLT, platelet; APTT, activated partial thromboplastin time; FS, full median sternotomy; MS, mini-sternotomy; IQR, interquartile range. *P*^a^, *p* value of FS group compared with MS group in overall series; *P*^b^, *p* value of FS group compared with MS group after propensity score-match.

### Study outcomes

After PSM, we compared the perioperative variables between the two groups. No statistically significant differences in CPB, aortic cross-clamp time, and operative time were found, but the MS group tends to have less CPB time (89.8 ± 42.7 vs. 94.2 ± 41.9, *P* = 0.078). Patients from the FS group had more 24-h (*P* < 0.001) and total drainage (*P* < 0.001); however, there are no significant differences in transfusion events including plasma, red blood cell (RBC), and platelets between the two groups. Compared with patients in the FS group, there were no significant more reoperation, intra-aortic balloon pump (IABP), hemofiltration, and re-intubated events in the MS group. The mechanical ventilation time (MVT), ICU stay, and postoperative stay in MS group are similar to those of FS group. Moreover, no significant difference between postoperative stroke and acute renal failure was found between the two groups. There were four in-hospital deaths in the FS group and the number became two after PSM, while there is no in-hospital death in the MS group (*P* = 0.211). Even with no significant difference, FS group tends to have more incision infections (four patients before PSM, one patient after PSM). Paravalvular leakage in the MS group was similar to that in the FS group (*P* = 0.459). Details are shown in Table 2.

**Table 2:** In-hospital outcomes in the overall series and in propensity score-matched pairs.

	Overall series			Propensity score-matched		
	FS group (*n* = 1247)	MS group (*n* = 198)	*P* ^a^	FS group (*n* = 198)	MS group (*n* = 198)	*P* ^b^
Mechanical valve	845 (67.8)	131 (66.2)	0.655	133 (67.2)	131 (66.2)	0.831
Operative time (min)	188.5 ± 112.9	212.6 ± 103.0	0.005	211.4 ± 79.8	212.6 ± 103.0	0.214
CPB (min)	95.8 ± 59.9	89.8 ± 42.7	0.172	94.2 ± 41.9	89.8 ± 42.7	0.078
Cross-clamp (min)	71.0 ± 34.3	72.8 ± 29.4	0.427	71.2 ± 30.9	72.8 ± 29.4	0.237
Re-clamp	5 (0.4)	0 (0.0)	0.478	1 (0.5)	0 (0.0)	0.460
Transfusion events						
Plasma >400 ml	66 (5.3)	11 (5.6)	0.865	12 (6.1)	11 (5.6)	0.830
RBC >5u	52 (4.2)	11 (5.6)	0.351	10 (5.1)	11 (5.6)	0.823
Platelets >2 u	76 (6.1)	14 (7.1)	0.634	15 (7.6)	14 (7.1)	0.847
24-hdrainage (ml)	198.5 ± 108.6	131.7 ± 82.8	<0.001	192.6 ± 91.2	131.7 ± 82.8	<0.001
Total drainage (ml)	832.8 ± 408.5	459.3 ± 306.3	<0.001	659.6 ± 298.7	459.3 ± 306.3	<0.001
Reoperation	0 (0.0)	0 (0.0)	—	0 (0.0)	0 (0.0)	—
IABP	4 (0.3)	0 (0.0)	0.554	1 (0.5)	0 (0.0)	0.500
Hemofiltration	4 (0.3)	0 (0.0)	0.554	2 (1.0)	0 (0.0)	0.499
Re-intubated	4 (0.3)	0 (0.0)	0.554	0 (0.0)	0 (0.0)	—
MVT (h)	9.7 (IQR 6.2–14.8)	9.4 (IQR 5.4–15.6)	0.609	9.8 (IQR 6.1–14.4)	9.4 (IQR 5.4–15.6)	0.284
Postoperative stroke	3 (0.2)	0 (0.0)	0.642	0 (0.0)	0 (0.0)	—
Acute renal failure	46 (3.7)	5 (2.5)	0.535	6 (3.0)	5 (2.5)	0.760
ICU stay (h)	48 (IQR 24–72)	48 (IQR 24–72)	0.931	48 (IQR 24–72)	48 (IQR 24–72)	0.637
Hospital cost	11.6 ± 5.1	11.3 ± 4.6	0.396	12.1 ± 6.5	11.3 ± 4.6	0.487
Paravalvular leakage	11 (0.9)	5 (2.5)	0.060	2 (1.0)	5 (2.5)	0.224
Incision infection	4 (0.3)	0 (0.0)	0.554	1 (0.5)	0 (0.0)	0.500
Death	4 (0.3)	0 (0.0)	0.554	2 (1.0)	0 (0.0)	0.499
Postoperative stay (days)	7.6 ± 3.4	7.6 ± 4.0	0.936	7.2 ± 3.2	7.6 ± 4.0	0.342

CPB, cardiopulmonary bypass; MVT, mechanical ventilation time; IABP, intra-aortic balloon pump; ICU, intensive care unit; FS, full median sternotomy; MS, mini-sternotomy; RBC, red blood cell; IQR, interquartile range. *P*^a^, *p* value of FS group compared with MS group in overall series; *P*^b^, *p* value of FS group compared with MS group after propensity score-match.

In aortic valve regurgitations patients, significantly less postoperative 24-h (129.2 ± 87.3 vs. 195.4 ± 98.5, *P* < 0.001) and total drainage (429.3 ± 216.5 vs. 694.8 ± 208.5, *P* < 0.001) are found in the mini-sternotomy group compared with full sternotomy group. In aortic stenosis patients, the results are similar (24-h drainage: 142.6 ± 79.8 vs. 188.3 ± 91.2, *P* < 0.001; total drainage: 472.3 ± 244.6 vs. 622.4 ± 214.5, *P* < 0.001). Table 3 shows these details.

**Table 3:** In-hospital outcomes in propensity score-matched pairs for aortic valve regurgitation and aortic stenosis subgroup.

	AR			AS		
	FS group (*n* = 108)	MS group (*n* = 123)	*P* ^a^	FS group (*n* = 90)	MS group (*n* = 75)	*P* ^b^
Operative time (min)	208.6 ± 112.0	196.1 ± 103.9	0.085	212.7 ± 82.4	237.5 ± 98.2	0.970
CPB (min)	87.6 ± 31.2	85.7 ± 44.5	0.742	103.3 ± 47.3	96.5 ± 38.5	0.327
Cross-clamp (min)	62.9 ± 21.9	60.8 ± 30.4	0.604	79.9 ± 30.8	71.6 ± 26.7	0.072
Re-clamp	0 (0.0)	0 (0.0)	—	1 (1.1)	0 (0.0)	0.545
Transfusion events						
Plasma >400 ml	7 (6.5)	6 (4.9)	0.598	5 (5.6)	5 (6.7)	0.508
RBC >5 u	6 (5.6)	6 (4.9)	0.817	4 (4.4)	5 (6.7)	0.733
Platelets >2u	8 (7.4)	9 (7.3)	0.979	7 (7.8)	5 (6.7)	0.784
24-hdrainage (ml)	195.4 ± 98.5	129.2 ± 87.3	<0.001	188.3 ± 91.2	142.6 ± 79.8	<0.001
Total drainage (ml)	694.8 ± 208.5	429.3 ± 216.5	<0.001	622.4 ± 214.5	472.3 ± 244.6	<0.001
Reoperation	0 (0.0)	0 (0.0)	—	0 (0.0)	0 (0.0)	—
IABP	0 (0.0)	0 (0.0)	—	1 (1.1)	0 (0.0)	0.545
Hemofiltration	1 (0.9)	0 (0.0)	0.468	1 (1.1)	0 (0.0)	0.545
Re-intubated	0 (0.0)	0 (0.0)	—	0 (0.0)	0 (0.0)	—
MVT (h)	9.7 (IQR 5.4–14.9)	9.5 (IQR 5.2–16.2)	0.647	9.8 (IQR 6.1–15.7)	9.2 (IQR 5.1–16.2)	0.466
Postoperative stroke	0 (0.0)	0 (0.0)	—	0 (0.0)	0 (0.0)	—
Acute renal failure	3 (2.8)	2 (1.6)	0.667	4 (4.4)	3 (4.0	0.656
ICU stay (h)	48 (IQR 24–72)	48 (IQR 24–72)	0.945	48 (IQR 24–72)	48 (IQR 24–72)	0.741
Hospital cost	12.2 ± 3.6	11.0 ± 5.3	0.087	12.2 ± 5.3	11.6 ± 3.1	0.432
Paravalvular leakage	2 (1.9)	4 (3.3)	0.471	0 (0.0)	1 (1.3)	0.455
Incision infection	1 (0.9)	0 (0.0)	0.468	0 (0.0)	0 (0.0)	—
Death	0 (0.0)	0 (0.0)	—	2 (2.2)	0 (0.0)	0.296
Postoperative stay (days)	8.7 ± 4.1	7.7 ± 4.6	0.132	8.0 ± 3.6	7.6 ± 2.7	0.427

AR, aortic valve regurgitation; AS, aortic stenosis; CPB, cardiopulmonary bypass; MVT, mechanical ventilation time; IABP, intra-aortic balloon pump; ICU, intensive care unit; FS, full median sternotomy; MS, mini-sternotomy; RBC, red blood cell; IQR, interquartile range. *P*^a^, *p* value of FS group compared with MS group in overall series; *P*^b^, *p* value of FS group compared with MS group after propensity score-match.

## Discussion

Patients are increasingly aware of minimally invasive options for cardiac surgery. However, concerns have been raised that aortic valve replacement with mini-incision may result in longer operative, CPB, and aortic clamp time, increasing the risk of postoperative adverse events (12, 13). In this case, the benefits of cosmesis and less bleeding might not be worth the increased risk of complications. Even though some studies reported that compared with standard full median sternotomy, mini-sternotomy has shown excellent outcomes in terms of postoperative complications and hospital stay, controversies still exist. Our study weighted by propensity-matching methods compared clinical outcomes between MS and FS for aortic valve replacement and confirmed that the mini-sternotomy procedure is safe, and associated with low perioperative complications.

In general, minimal access valve operations mean a reduction of working space and poor exposure, which make a large technical challenge, reflecting the longer operative, aortic cross-clamp, and CPB times. Nair et al. reported the results of an randomized controlled trial (RCT) study, which shows aortic cross-clamp and CPB times were significantly prolonged by MS (14). That has raised some concerns regarding the safety of mini-sternotomy especially in high-risk patients, for the longer CPB and cross-clamp times might increase the risk of perioperative complications. Our study indicates that there are no significant differences in operation, aortic cross-clamp, and CPB times between MS and FS group. Since 2004, minimal access valve operations have been made in our centers and many surgeons have completed training in the mini-incision technique. Even for some senior surgeons, less operative time in MS aortic valve replacement than FS approach because only half of the manubrium and shorter incision need to be closed. In this study, no mini-incisions were converted to full sternotomy.

A retrospective propensity-matched analysis of data concluded that MS is safe and does not increase the risk of postoperative complications (15). Even two meta-analyses concluded that aortic valve replacement with mini-incision approaches is superior in certain aspects of postoperative recovery (16, 17). Our study shows similar results that compared with patients in the FS group, no significant more IABP, hemofiltration, postoperative stroke, acute renal failure, and in-hospital death are found in the MS group. Our result shows less postoperative drainage in the MS group, which is likely due to the smaller mediastinal dissection required for the less invasive approach. Some similar results had been reported (6, 18). To prevent bias, we analyzed patient classification, and in either aortic valve regurgitations or aortic stenosis subgroup, patients with mini-sternotomy have significantly less postoperative 24-h and total drainage compared to those with full sternotomy.

Generally, the rate of incision infection was higher in the patients who underwent full median sternotomy procedures, but in our study, only one patient in the FS group had incision infection and the difference was not statistically significant. In a word, this study shows no disadvantages of mini-sternotomy and less postoperative drainage. So, mini-sternotomy for aortic valve replacement is a safe procedure.

## Limitations

This study still has several limitations. It is based on a retrospective analysis of a single center; thus, it only reflects single-center experience. The decision as to whether to perform MS or FS was likely based on multiple factors that may not have been completely collected in this retrospective review. We used propensity score matching to reduce the impact of potential confounding factors. However, after matching, there were only 198 cases in each group. In the future, more cases from multicenter will be enrolled to confirm the conclusion.

## Data Availability

The original contributions presented in the study are included in the article/Supplementary Material, further inquiries can be directed to the corresponding author.

## References

[1] Rao PN Kumar AS. Aortic valve replacement through right thoracotomy. Tex Heart Inst J. (1993) 20:307–8. PMID: ; PMCID: 8298332 PMC325118

[2] Cosgrove DM Sabik JF. Minimally invasive approach for aortic valve operations. Ann Thorac Surg. (1996) 62:596–7. 10.1016/0003-4975(96)00418-3 8694642

[3] Von Segesser LK Westaby S Pomar J Loisance D Groscurth P Turina M. Less invasive aortic valve surgery: rationale and technique. Eur J Cardiothorac Surg. (1999) 15:781–5. 10.1016/S1010-7940(99)00119-0 10431859

[4] Ruttmann E Gilhofer TS Ulmer H Chevtchik O Kocher A Schistek R Propensity score-matched analysis of aortic valve replacement by mini-thoracotomy. J Heart Valve Dis. (2010) 19:606–14. PMID: 21053740

[5] Merk DR Lehmann S Holzhey DM Dohmen P Candolfi P Misfeld M Minimal invasive aortic valve replacement surgery is associated with improved survival: a propensity-matched comparison. Eur J Cardiothorac Surg. (2015) 47:11–7. 10.1093/ejcts/ezu068 24599160

[6] Furukawa N Kuss O Aboud A Schönbrodt M Renner A Meibodi KH Ministernotomy versus conventional sternotomy for aortic valve replacement: matched propensity score analysis of 808 patients. Eur J Cardiothorac Surg. (2014) 46:221–6. 10.1093/ejcts/ezt616 24446478

[7] Tabata M Umakanthan R Cohn LH Bolman RM 3rd Sheakar PS Chen FY Early and late outcomes of 1000 minimally invasive aortic valve operations. Eur J Cardiothorac Surg. (2008) 33:537–41. 10.1016/j.ejcts.2007.12.037 18255305

[8] Brown ML McKellar SH Sundt TM Schaff HV. Ministernotomy versus conventional sternotomy for aortic valve replacement: a systematic review and meta-analysis. J Thorac Cardiovasc Surg. (2009) 137:670–9. 10.1016/j.jtcvs.2008.08.010 19258087

[9] Brinkman WT Hoffman W Dewey TM Culica D Prince SL Herbert MA Aortic valve replacement surgery: comparison of outcomes in matched sternotomy and port access groups. Ann Thorac Surg. (2010) 90:131–5. 10.1016/j.athoracsur.2010.03.055 20609763

[10] Johnston DR Atik FA Rajeswaran J Blackstone EH Nowicki ER Sabik JR III Outcomes of less invasive J-incision approach to aortic valve surgery. J Thorac Cardiovasc Surg. (2012) 144:852–8. 10.1016/j.jtcvs.2011.12.008 22244556

[11] Shekar PS. Minimal access aortic valve surgery through an upper hemisternotomy approach. Oper Tech Thorac Cardiovasc Surg. (2010) 15:321–35. 10.1053/j.optechstcvs.2010.11.001

[12] Flameng W Herregods MC Hermans H Van der Mieren G Vercalsteren M Poortmans G Effect of sutureless implantation of the perceval S aortic valve bioprosthesis on intraoperative and early postoperative outcomes. J Thorac Cardiovasc Surg. (2011) 142:1453–7. 10.1016/j.jtcvs.2011.02.021 21474151

[13] Folliguet TA Laborde F Zannis K Ghorayeb G Haverich A Shrestha M. Sutureless perceval aortic valve replacement: results of two European centers. Ann Thorac Surg. (2012) 93:1483–8. 10.1016/j.athoracsur.2012.01.071 22541180

[14] Nair SK Sudarshan CD Thorpe BS Singh J Pillay T Catarino P Mini-stern trial: a randomized trial comparing mini-sternotomy to full median sternotomy for aortic valve replacement. J Thorac Cardiovasc Surg. (2018) 156(6):2124–32. 10.1016/j.jtcvs.2018.05.057 30075959

[15] Attia RQ Hickey GL Grant SW Bridgewater B Roxburgh JC Kumar P Minimally invasive versus conventional aortic valve replacement: a propensity-matched study from the UK national data. Innovations (Phila). (2016) 11:15–23. 10.1097/imi.0000000000000236 26926521 PMC4791314

[16] Lim JY Deo SV Altarabsheh SE Jung SH Erwin PJ Markowitz AH Conventional versus minimally invasive aortic valve replacement: pooled analysis of propensity-matched data. J Card Surg. (2015) 30:125–34. 10.1111/jocs.12493 25533177

[17] Phan K Xie A Di Eusanio M Yan TD. A meta-analysis of minimally invasive versus conventional sternotomy for aortic valve replacement. Ann Thorac Surg. (2014) 98:1499–511. 10.1016/j.athoracsur.2014.05.060 25064516

[18] Khoshbin E Prayaga S Kinsella J Sutherland FW. Mini-sternotomy for aortic valve replacement reduces the length of stay in the cardiac intensive care unit: meta-analysis of randomised controlled trials. BMJ Open. (2011) 1(2):e000266. 10.1136/bmjopen-2011-000266 22116090 PMC3225590

